# Outcomes of *Trypanosoma cruzi* and *Trypanosoma evansi* infections on health of Southern coati (*Nasua nasua*), crab-eating fox (*Cerdocyon thous*), and ocelot (*Leopardus pardalis*) in the Brazilian Pantanal

**DOI:** 10.1371/journal.pone.0201357

**Published:** 2018-08-15

**Authors:** Filipe Martins Santos, Gabriel Carvalho de Macedo, Wanessa Teixeira Gomes Barreto, Luiz Gustavo Rodrigues Oliveira-Santos, Carolina Martins Garcia, Guilherme de Miranda Mourão, Grasiela Edith de Oliveira Porfírio, Elizangela Domenis Marino, Marcos Rogério André, Lívia Perles, Carina Elisei de Oliveira, Gisele Braziliano de Andrade, Ana Maria Jansen, Heitor Miraglia Herrera

**Affiliations:** 1 Programa de Pós-Graduação em Ciências Ambientais e Sustentabilidade Agropecuária, Universidade Católica Dom Bosco, Campo Grande, Mato Grosso do Sul, Brazil; 2 Programa de Pós-Graduação em Ecologia e Conservação, Universidade Federal de Mato Grosso do Sul, Campo Grande, Mato Grosso do Sul, Brazil; 3 Laboratório de Vida Selvagem, Centro de Pesquisa Agropecuária do Pantanal, Empresa Brasileira de Pesquisa Agropecuária, Corumbá, Mato Grosso do Sul, Brazil; 4 Universidade Estadual Paulista, Faculdade de Ciências Agrárias e Veterinárias, Jaboticabal, São Paulo, Brazil; 5 Laboratório de Biologia de Tripanosomatídeos, Instituto Oswaldo Cruz, Fundação Oswaldo Cruz, Rio de Janeiro, Rio de Janeiro, Brazil; Universidade Federal de Minas Gerais, BRAZIL

## Abstract

The occurrence of *Trypanosoma* spp. in wild carnivore populations has been intensively investigated during the last decades. However, the impact of these parasites on the health of free-living infected animals has been largely neglected. The Pantanal biome is the world’s largest seasonal wetland, harboring a great diversity of species and habitats. This includes 174 species of mammals, of which 20 belong to the order Carnivora. The present study aimed to investigate the effect of *Trypanosoma evansi* and *Trypanosoma cruzi* infections and coinfections on the health of the most abundant carnivores in the Pantanal: coati (*Nasua nasua*), crab-eating fox (*Cerdocyon thous*), and ocelot (*Leopardus pardalis*). We captured 39 coatis, 48 crab-eating foxes, and 19 ocelots. Diagnostic tests showed *T*. *cruzi* infection in 7 crab-eating foxes and 5 coatis. Additionally, 7 crab-eating foxes, 10 coatis, and 12 ocelots were positive for *T*. *evansi*. We observed coinfections in 9 crab-eating foxes, 8 coatis, and 2 ocelots. This is the first report of *T*. *evansi* and *T*. *cruzi* infection on the health of free-living ocelots and crab-eating foxes. We showed that single *T*. *evansi* or *T*. *cruzi* infection, as well as coinfection, caused some degree of anemia in all animals, as well as an indirect negative effect on body condition in coatis and crab-eating foxes via anemia indicators and immune investment, respectively. Furthermore, the vigorous immune investment observed in sampled coatis, crab-eating foxes and ocelots infected by *T*. evansi, *T*. *cruzi* and coinfected can be highly harmful to their health. Overall, our results indicate that single and combined infection with *T*. *evansi* and *T*. *cruzi* represent a severe risk to the health of wild carnivores in the Pantanal region.

## Introduction

Although parasites are known to use resources from their hosts, thus affecting their energy balance [1, 2], little is known about the effect of parasitism on the health of mammals that inhabit natural environments [3]. Loss of physical condition due to parasitism can negatively influence reproductive rates, movement, and survival of infected hosts [4–6].

The occurrence of *Trypanosoma* spp. in wild carnivores has sparked intensive investigation worldwide in the last decades [7–11]. Nevertheless, the impact of these parasites on the health of free-living infected animals has been largely neglected. Studies documenting the outcomes of *Trypanosoma cruzi* and *T*. *evansi* on the health of wild carnivores have shown that these parasites can cause damage to the health of their hosts [12, 13].

In enzootic areas *Trypanosoma* species are maintained in a complex network transmission cycles including mammals and blood sucking vectors. While *T*. *evansi* transmission occur mechanically by hematophagus flies, such as tabanids and *Stomoxys calcitrans*, *T*. *cruzi* is cyclically transmitted through Triatominae faces [14,15].

Natural infection by *T*. *evansi* causes different degrees of anemia in several domestic and wild mammals. Infected animals display widespread subcutaneous edema, fever, lethargy, weight loss, abortion, nasal and ocular bleeding, and stiffness of the pelvic members [13,16,17]. Additionally, coatis and golden lion tamarins (*Leontopithecus rosalia*) infected with *T*. *cruzi* have been reported to present hematological disorders and cardiac diseases, respectively [18, 19, 20].

It has been suggested that coinfections play a central role in driving parasite dynamics [21, 22]. One such case is natural infection of the golden lion tamarin with *T*. *cruzi*: higher parasitemia was observed in animals coinfected with nematode worms [23]. In fact, coinfections with multi-host parasites in free-living mammals may be highly dynamic and unpredictable because the ecological processes are stochastic. Cooccurrence of *T*. *cruzi* and *T*. *evansi* in wild carnivores (e.g. *Cerdocyon thous*, *Leopardus pardalis* and *Nasua nasua*) has already been recorded [8, 13], but there is no knowledge about the impact of these parasites on the hosts’ health.

Given the scarce knowledge about the health of free-living neotropical mammals infected by trypanosomatids, the present study aimed to investigate the effect of single and combined infection with *T*. *evansi* and *T*. *cruzi* on the hematological parameters of coatis (*N*. *nasua*), crab-eating foxes (*C*. *thous*), and ocelots (*L*. *pardalis*) in the Pantanal biome, the largest floodplain of the world.

## Methodology

### Study area

The study was carried out in a private ranch in the sub-region of Nhecolândia (19° 8'31.71"S 56°47'40.97"O). The soil is sandy and vegetation is composed of deciduous and semi-deciduous forests in “cordilheiras” (long strings of forest) and "capões" (forest patches surrounded by open native grasslands). The climate is marked by two distinct seasons: a warm rainy period (October to March) and a cold dry period (April to September). About 174 species of mammals was recorded in the Pantanal, of which 20 belongs to the order Carnivora [24]. Among them, three species are considered abundant in the Pantanal: the southern coati, the crab-eating fox, and the ocelot [25].

### Sample collection

Carnivores were sampled from November 2015 to October 2016. We used 50 Box-traps (90 × 45 × 50; EquiposFauna^®^) baited with bacon and tinned sardines to capture the target species. Once trapped, animals were sedated with an intramuscular injection of Zoletil 50 (containing tiletamine hydrochloride and zolazepan hydrochloride; Virbac) respecting the dosages currently recommended for each species, and marked with subcutaneous transponders (AnimalTag^®^). Body condition (body size and mass) were recorded. Blood (~4 mL) was collected from the jugular vein, placed in tubes with and without ethylenediamine tetraacetic acid (EDTA), and stored in cool boxes until laboratory procedures. The animals were released at the capture site after recovery from anesthesia.

### Ethical approval

All field procedures were conducted in accordance with a license granted by the Biodiversity Information and Authorization System of the Chico Mendes Institute for Biodiversity Conservation (license number 49662–5). The present study was approved by the Ethics Committee for Animal Use of Dom Bosco Catholic University, Campo Grande, MS (license number 19/2015).

### Health parameters

The health of carnivores was inferred, mainly, by means of blood parameters. Packed cell volume (PCV), red blood cell counts (RBC), and white blood cell counts (WBC) were measured up to 8 h after blood collection in Neubauer chambers, as described by Voigt [26]. Mean corpuscular volume (MCV) was calculated based on the RBC and PCV values. The immunoglobulin concentration (IgG) was determined by titration with the indirect fluorescent antibody test (IFAT) [27,28] and by optical density using enzyme-linked immunosorbent assay (ELISA)[29]. Leukocyte (eosinophils, lymphocytes, monocytes, and neutrophils) counts were performed using blood smears fixed with methanol and stained with Giemsa [30].

We evaluated the health condition of sampled carnivores in terms of: (a) PCV, RBC, and MCV as anemia indicators; (b) monocyte and neutrophil counts as indicators of infection responses; and (c) lymphocyte counts and IgG concentration as indicators of immune investment [30].

### Diagnosis of *T*. *evansi* and *T*. *cruzi* infection

Infections with *T*. *evansi* and *T*. *cruzi* were assessed by parasitological, molecular and serological tests. The parasitological test for *T*. *evansi* used the microhematocrit centrifuge technique (MHCT) according to Woo [31]. The absence of kinetoplast in buffy coat smears confirms *T*. *evansi*. For *T*. *cruzi*, the test was based on hemoculture by inoculating 300 μL of blood in Novy McNeal Nicole (NNN) medium with liver infusion tryptose (LIT), in duplicate. Hemoculture tubes were incubated at 27 °C for 30 days and monitored once a week.

Molecular detection of *Trypanosoma* spp. infection was performed by nested polymerase chain reaction (nPCR). Genomic DNA was extracted from 200 μL of blood with EDTA using the QIAamp Blood DNA Mini Kit (Qiagen) according to the manufacturer’s instructions. Total DNA was diluted with 50 μL elution buffer and stored at -20 °C until molecular diagnosis. We used as a target a variable region of the trypanosome 18S rRNA gene (600 bp), with external primers TRY927F and TRY927R, and internal primers SSU561F and SSU561R, according to Smith et al. [32]. TBR1 and TBR2 primers were applied to positive 18S rRNA samples to amplify a sequence of mini-chromosome satellite DNA for *T*. *evansi*, according to Masiga et al. [33]. Furthermore, D71 and D72 primers were used to amplify a conserved sequence of the large subunit of the ribosomal DNA gene (24Sα rDNA) in *T*. *cruzi*, according to Souto and Zingales [34]. Each reaction included sterile distilled water instead of DNA as negative control, and positive control samples from *T*. *cruzi* and *T*. *evansi* strains. PCR products were visualized in 2% agarose gel after ethidium bromide staining under ultraviolet light.

Serological tests were used to detect anti-*T*. *evansi* IgG antibodies in crab-eating foxes and ocelots by IFAT using a commercial fluorescein-conjugated antibody against dogs and cats IgGs, respectively. The cut-off value for IFAT was 1:40 [27]. There is presently no fluorescein-conjugated antibody against coatis’ IgGs. To detect anti-*T*. *cruzi* IgG antibodies, we used IFAT (fluorescein-conjugated antibody against dogs and cats IgGs) and ELISA (fluorescein-conjugated antibody against raccoon’s IgGs), as described by Rocha et al. [28] and Alves et al. [29], respectively. The cut-off value for ELISA was defined as the mean optical absorbance of the negative controls +20%. We added two positive and two negative control sera to each reaction plate, as described by Alves et al. [29].

We considered an animal to be positive to *Trypanosoma* infection, when any of the four diagnostic tests used (hemoculture, MHCT, PCR or/and serological tests) was positive.

### Data analysis

Descriptive statistic (mean ± standard deviation) was applied to obtain the mean health parameters of the specimens. The Shapiro-Wilk test served to establish whether the distribution was normal. Finally, a Kruskal-Wallis test was applied to determine the differences between: no infection, *T*. *evansi* infection, *T*. *cruzi* infection, and coinfection. *Post hoc* Mann-Whitney tests were used to assess pair-wise results of the Kruskal-Wallis test.

To determine the direct and indirect influences of infections and coinfections in relation to anemia, infection responses, immune investment and body condition, we carried out a path analysis. We assessed variation in body condition based on the standardized residuals from an ordinary linear regression between body mass (g) and head-body length (mm) of individuals, while accounting for age and sex effects (13). This should circumvent the effects of animal growth on the condition index. Therefore, the residuals were calculated for males and females separately. To perform dimensionality reduction of anemia, infection responses, and immune investment values, we used the principal coordinate analysis, a geometric technique that converts a matrix of distances between points in multivariate space into a projection that maximizes the amount of variation along a series of orthogonal axes. We used an r value ≥ 0.60 to interpret the results (positive or negative effect) of the path analysis.

Path analysis describes two types of effects: direct and indirect. When the exogenous variable has an arrow directed towards the dependent variable, the effect is direct. When the effect is indirect, the arrow crosses one or more than one dependent variable until the final effect. The variables were considered to be statistically significant for *p* values ≤ 0.05. All data were analyzed using R (version 3.4.2) [35].

## Results

We sampled 106 adult carnivores: 39 coatis (17 females and 22 males), 48 crab-eating foxes (22 females and 26 males), and 19 ocelots (eight females and 11 males). The different diagnostic tests showed *T*. *evansi* positivity in 12 ocelots (4 females and 8 males), 10 coatis (6 females and 4 males) and 7 crab-eating foxes (1 female and 6 males). Additionally, we found *T*. *cruzi* infection in 7 crab-eating foxes (4 female and 3 males), and 5 coatis (5 males). We observed coinfection in 9 crab-eating foxes (4 females and 5 males), 8 coatis (3 females and 5 males), and 5 ocelots (3 females and 2 males) (Table 1).

**Table 1:** Number of positive coatis (*Nasua nasua)*, crab-eating-foxes (*Cerdocyon thous*) and ocelots (*Leopardus pardalis*) for *Trypanosoma cruzi* and *Trypanosoma evansi* in the Pantanal. Samples were collected from November 2015 up to October 2016.

Infection	Diagnostic Test	*N*. *nasua* (n = 39)	*C*. *thous* (n = 48)	*L*. *pardalis* (n = 19)
*T*. *cruzi*	HC	03 (8)	-	-
	PCR	06 (15)	-	-
	Serological tests	11 (28)	16 (33)	05 (26)
*T*. *evansi*	MHCT	10 (26)	-	-
	PCR	18 (46)	10 (21)	12 (63)
	Serological tests	ND	7 (15)	15 (79)
Coinfection	HC/MHCT	-	-	-
	PCR	03 (08)	-	-
	Serological tests	-	03 (06)	04 (21)

The data are expressed by number of captured animals/relative abundance (%). (−) negative results. ND: Not Done, HC: Hemoculture, PCR: Polymerase Chain Reaction, MHCT: Microhematocrit Centrifuge Technique

### Coatis

Mean PCV values was significantly lower (χ2 = 11.94, df = 03, *p* < 0.05) in coatis infected with *T*. *evansi* (28.5±4.9) (*U* = 34.5, *p* < 0.05) and in coinfected animals (26.4±7.8) (*U* = 23, *p* < 0.05), when compared with non-infected coatis (35.8±5.2). Moreover, *T*. *evansi* infected and coinfected animals also presented lower means of RBC (*T*. *evansi* infection: 3.3×10^6^ ±1.7 and coinfection: 3.3×10^6^±1.2) and MCV (*T*. *evansi* infection: 94.2±19.7 and coinfection: 86.4±31.8) values, however without statistical significance (RBC: χ2 = 3.015, df = 03, *p* > 0.05; MCV: χ2 = 0.9674, df = 03, *p* > 0.05). Mean leukocyte values (χ2 = 11.07, df = 03, *p* < 0.05) were significantly higher in *T*. *cruzi*-infected (27,150±8,427) (*U* = 6.5, *p* < 0.05) and coinfected coatis (27,719±7,750) (*U* = 26, *p* < 0.05) (Table 2).

**Table 2:** Hematological mean values of coatis (*Nasua nasua*) infected with *Trypanosoma evansi* (TE), *Trypanosoma cruzi* (TC), and in coinfected (TE/TC) animals in the sub-region of Nhecolândia, Pantanal, between November 2015 and October 2016.

*Nasua nasua*	Non infected (n = 16)	TE positive (n = 10)	TC positive (n = 05)	TE/TC positive (n = 08)
RBC	4.2±1.6^a^	3.3±1.7^a^	3.9±1.1^a^	3.3±1.2^a^
PCV	35.8±5.2^a^	28.5±4.9^b^	36.4±6.9^a^	26.4±7.8^b^
MCV	97.7±44.7^a^	94.2±19.7^a^	98±34.3^a^	86.4±31.8^a^
WBC	18,212±9,359^a^	15,595±6,297^a^	27,150±8,427^b^	27,719±7,750^b^
Eosinophils	663±497^a^	444±310^a^	1,333±1,248^b^	1,681±1,013^b^
Lymphocytes	3,785±4,739^a^	1,477±360^a^	2,474±2,122^b^	3,708±1,173^c^
Monocytes	868±843^a^	997±486^a^	2,083±1,269^b^	2,260±1,068^b^
Neutrophils	12,362±6,031^a^	12,414±6,035^a^	19,624±5,768^b^	19,816±6,086^b^

Different letters denote statistical significance (*p* < 0.05). PCV: packed cell volume; RBC: red blood cell counts (×10^6^); WBC: white blood cell counts; MCV: mean corpuscular volume.

Path analysis showed a negative direct effect of *T*. *evansi* infection (path coefficient = -0.30, *p* < 0.05) on anemia indicators, resulting in lower PCV (r = 0.84) and MCV (r = 0.65). Although we did not observe a direct effect on anemia indicators of coatis infected with *T*. *cruzi*, we found an increased negative direct effect on these values in coinfected animals (path coefficient = -0.47, *p* < 0.05). Additionally, our results showed that *T*. *evansi* infection had a negative influence on body condition via anemia indicators (path coefficient = 0.37, *p* < 0.05). Moreover, this effect was potentiated in coinfected animals (S1 Fig).

We observed that *T*. *cruzi* positively affected infection responses (path coefficient = 0.44, *p* < 0.05). In contrast, *T*. *evansi* alone was unable to alter the infection response, but exhibited an increased effect (path coefficient = 0.52, *p* < 0.05) in coinfected animals, resulting in more monocytes (r = 0.60) and neutrophils (r = 0.89) (S2 Fig).

We observed that *T*. *cruzi* (path coefficient = -0.43, *p* < 0.05) infection had a positive effect on immune investment, increasing further in coinfected animals (path coefficient of = -0.61, *p* < 0.05; path coefficient = -0.46, *p* < 0.05). This resulted in increased numbers of lymphocytes (r = -0.60; r = -0.70) and anti-*T*. *cruzi* IgGs (r = -0.79; r = -0.60) (S3 Fig). Additionally, *T*. *cruzi*, *T*. *evansi*, and coinfection with both parasites had also indirect negative effects on body condition via immune investment (path coefficient = -0.34, *p* < 0.05) (S3 Fig).

### Crab-eating foxes

No significant differences were observed for mean RBC (χ2 = 0.3187, df = 03, *p* > 0.05), PVC (χ2 = 2.552, df = 03, *p* > 0.05), and MCV (χ2 = 0.5056, df = 03, *p* > 0.05) values between infected and non-infected crab-eating foxes. Nevertheless, our data indicated a minor decrease of these values for *T*. *evansi*-infected animals. Furthermore, we observed a significant increase in WBC mean values (χ2 = 6.036, df = 03, *p* < 0.05) in *T*. *cruzi*-infected animals due to neutrophilia (10,823±4,745) (*U* = 40, *p* < 0.05) (Table 3).

**Table 3:** Hematological mean values for crab-eating foxes (*Cerdocyon thous*) infected with *Trypanosoma evansi* (TE), *Trypanosoma cruzi* (TC), and in coinfected (TE/TC) animals in the sub-region of Nhecolândia, Pantanal, between November 2015 and October 2016.

*Cerdocyon thous*	Non infected (n = 25)	TE positive (n = 07)	TC positive (n = 07)	TE/TC positive (n = 09)
RBC	3.1±1.3^a^	3±0.4^a^	3.2±1^a^	3±1^a^
PCV	38.1±7.9^a^	37.7±3.7^a^	45.6±19.3^a^	40±3.6^a^
MCV	143.4±66.1^a^	127.1±26.9^a^	154.4±73.2^a^	144.9±45.1^a^
WBC	10,424±4,491^a^	12,428±6,897^a^	14,764±5,528^b^	12,905±3,563^a^
Eosinophils	852±479^a^	1,299±1,303^a^	881±541^a^	1,048±1,046^a^
Lymphocytes	2,006±1,318^a^	1,747±1,351a	2,138±1,118^a^	2,286±1,256^a^
Monocytes	778±336^a^	686±450^a^	874±577^a^	780±517^a^
Neutrophils	6,785±4,068^a^	8,638±5,429^a^	10,823±4,745^b^	8,677±3,547^a^

Different letters denote statistical significance (*p* < 0.05). PCV: packed cell volume; RBC: red blood cell counts (×10^6^); WBC: white blood cell counts; MCV: mean corpuscular volume.

Path analysis revealed that infections with *T*. *cruzi* and *T*. *evansi* had no effect on anemia indicators of crab-eating foxes. However, we found a negative effect on the infection responses following *T*. *cruzi* infection (path coefficient = 0.27, *p* < 0.05) and coinfection (path coefficient = 0.26, *p* < 0.05), resulting in fewer monocytes (r = -0.62) and neutrophils (r = -0.64) (S4 Fig).

Moreover, we observed that *T*. *cruzi* (path coefficient = -0.72, *p* < 0.05) infection and coinfection (path coefficient = -0.79, *p* < 0.05) had a positive influence on immune investment, as manifested by an increase in anti-*T*. *cruzi* IgGs (r = -0.97) (S5 Fig).

Finally, we observed that *T*. *evansi* infection (path coefficient = 0.31, *p* < 0.05) and coinfection (path coefficient = 0.31, *p* < 0.05; path coefficient = 0.52, *p* < 0.05) influenced positively immune investment resulting in more lymphocytes (r = 0.70) and anti-*T*. *evansi* IgGs (r = 0.72 r = 0.60) (S6 Fig). Both, *T*. *evansi* infection and coinfection, had an indirect negative effect on body condition via immune investment on lymphocytes and anti-*T*. *evansi* IgGs (path coefficient = -0.41, *p* < 0.05; path coefficient = -0.56, *p* < 0.05) (S6 Fig).

### Ocelots

Although we found lower RBC and PCV mean values for ocelots parasitized by *T*. *evansi* and in those coinfected with both parasites than in non-infected animals, differences were not statistically significant (RBC: χ2 = 3.672, df = 03, *p* > 0.05; PVC: χ2 = 5.12, df = 03, *p* > 0.05; and MCV: χ2 = 0.2237, df = 03, *p* > 0.05) between non infected and *T*. *evansi*-infected ocelots (Table 4).

**Table 4:** Hematological mean values among ocelots (*Leopardus pardalis)* infected with *Trypanosoma evansi* (TE) and coinfected with *T*. *evansi*/*Trypanosoma cruzi* (TE/TC) in the sub-region of Nhecolândia, Pantanal, between November 2015 and October 2016.

*Leopardus pardalis*	Non infected (n = 02)	TE positive (n = 12)	TE/TC positive (n = 05)
RBC	6.9±1.9^a^	4.5±1.8^a^	4.1±1.1^a^
PCV	41.9±4.2^a^	32.1±4.7^a^	32.9±3.6^a^
MCV	83±4.16^a^	81.5±34.3^a^	85.8±18.7^a^
WBC	17,275±16,723^a^	14,650±4,479^a^	16,830±5,357^a^
Eosinophils	54±77^a^	178±312^a^	331±161^a^
Lymphocytes	1,545±1,107^a^	2,372±1,470^a^	2,493±907^a^
Monocytes	600±386^a^	874±704^a^	1,229±796^a^
Neutrophils	15,048±15,346^a^	11,027±3,513^a^	12,900±4,318^a^

Different letters denote statistical significance (*p* < 0.05). PCV: packed cell volume; RBC: red blood cell counts; WBC: white blood cell counts; MCV: mean corpuscular volume.

Path analysis showed that *T*. *evansi* (path coefficient = 0.90, *p* < 0.05) and coinfection (path coefficient = 0.73, *p* < 0.05) resulted in lower PCV (r = -0.93) values (S7 Fig). Moreover, the decrease in body condition was influenced by fewer RBC (r = -0.91) and higher MCV (r = 0.88) values, irrespective of infection with *T*. *evansi* or coinfection (path coefficient = -0.41, *p* < 0.05) (S7 Fig). Infection with *T*. *cruzi* and *T*. *evansi* did not have any effect on the infection response in ocelots.

Furthermore, we observed that coinfection (path coefficient = -1.03, *p* < 0.05) had a positive effect on immune investment, marked by an increase in anti-*T*. *cruzi* IgGs (r = -0.97) (S8 Fig). A similar positive effect on immune investment was observed also with *T*. *evansi* infection (path coefficient = -0.99, *p* < 0.05; path coefficient = 0.94, *p* < 0.05) and coinfection (path coefficient = -0.77, *p* < 0.05; path coefficient = 0.96, *p* < 0.05) (S8 Fig). This resulted in more lymphocytes (r = 0.75) and higher anti-*T*. *evansi* IgG (r = -0.87) values (S9 Fig). Additionally, *T*. *evansi* infection and coinfection had indirect positive effects on body condition via immune investment (path coefficient = 0.59, *p* < 0.05) (S9 Fig).

## Discussion

Our results reveal that *T*. *evansi* infection in coatis, crab-eating foxes and ocelots causes some degree of anemia. Anemia has been recorded previously in coatis infected with *T*. *evansi* [13, 17–19], but the present study is the first report of *T*. *evansi* infection resulting in anemia also in free-living ocelots and crab-eating foxes. Anemia is characteristic of *T*. *evansi* infections [17, 36–38] and can represent a threat to the health of carnivores in the Pantanal wetland, as suggested by infection rates of 89% (17/19) in ocelots, 46% (18/39) in coatis, and 33% (16/48) in crab-eating foxes.

Even though *T*. *cruzi* infection could not induce anemia in coatis, coinfection with *T*. *evansi* caused the degree of anemia to become more severe, a finding previously observed by Olifiers et al. 2015 [13]. The microcytic hypochromic anemia, characterized by the low MCV values in *T*. *evansi*-infected coatis and the even lower values in coinfected animals, may correlate to deficient hemoglobin synthesis due to iron deficiency [39–41], as observed in *T*. *evansi* infections [42, 43]. The low MCV values could also result from the influx of iron into the cell, which is necessary for the multiplication of intracellular amastigote forms of *T*. *cruzi* [44, 45].

Anemia was observed also in ocelots, as suggested by small differences in anemia indices in animals infected with *T*. *evansi* and coinfected with *T*. *cruzi*, as well as through direct effect of *T*. *evansi* infection and coinfection on PCV values tested by path analysis. Anemia has been recorded previously in domestic cats experimentally infected with *T*. *evansi* [46–48].

Moreover, lower RBC and higher MCV values indicated a megaloblastic anemia, which negatively influenced ocelots’ body condition, irrespective of *Trypanosoma* spp. infection. Although we have not investigated other pathogens or other causes, in natural environments animals are constantly and concomitantly exposed to a variety of parasites, particularly *Anaplasma* spp., *Mycoplasma* spp., and piroplasmids, which cause lysis in parasited red blood cells and the consequent drop in RBC values. The same parasites have been described to infect ocelots in the studied area [49–51]. Additionally, the observed increase in MCV values may have metabolic origin and be associated with deficiency of vitamin B12, which is found mainly in protein diets, or/and in hepatic dysfunction.

Regarding crab-eating foxes, we observed a slight decrease in indicators of anemia only in *T*. *evansi*-infected animals. Importantly, domestic dogs that have been experimentally or naturally infected with *T*. *evansi* display evident signs of anemia and the course of infection is fatal if not treated [52, 53]. Therefore, free-living crab-eating foxes parasitized with *T*. *evansi* may become sick and prostrate, consequently they may die or are not collected.

We observed discrete leucopenia due to fewer lymphocytes and eosinophils in coatis parasited with *T*. *evansi*. Immunosuppression in coatis infected with *T*. *evansi* has been described previously in natural and experimental studies [13, 18, 19, 54]. This phenomenon varies in nature due to different communities of parasites in their hosts, as well as the influence of marked seasonality of resources, which is characteristic of the Pantanal region [12, 27].

The leukocytosis observed here in *T*. *cruzi*-infected and coinfected coatis is typical of the acute phase of *T*. *cruzi* infection [55]. The increase in leucocytes during *T*. *cruzi* infection in wild mammals has been reported in *Thrychomis pachyurus* and coatis under experimental and natural conditions, respectively [12, 56].

We observed a notable infection response in coatis infected with *T*. *cruzi* and in coinfected animals. Monocytosis is a sign of immune response during the acute phase of *T*. *cruzi* infection [57, 58]. Throughout the chronic phase of *T*. *cruzi* infection, neutrophils act together with monocytes and lymphocytes to repair the tissue damage caused by *T*. *cruzi* amostigote [59]. An increase in monocyte and neutrophil values is an important hallmark of infection by *T*. *cruzi* in naturally infected coatis, as already reported by Martínez-Hernández et al. 2016 [12].

We observed a decrease in lymphocytes in crab-eating foxes infected with *T*. *evansi*, confirming the findings of Da Silva et al. 2011 [60] in the chronic phase of *T*. *evansi* infection in laboratory rodents. Indeed, domestic dogs naturally and experimentally infected with *T*. *evansi* displayed fewer WBCs and neutrophils [52, 53]. Additionally, the decrease in monocytes and neutrophils observed in crab-eating foxes infected with *T*. *cruzi* or in coinfected animals, was similar to that reported in dogs during the early stages of *T*. *cruzi* experimental infection [61]. Such immunosuppression, even if transient, can impair the health of the animal.

An increase in immune investment in coatis, ocelots, and crab-eating foxes infected with *T*. *cruzi*, *T*. *evansi*, or in coinfected animals recorded in the present study may be associated with a potent stimulation of cellular and humoral immune response, characteristic of trypanosome infection [62–64]. The strong production of immunoglobulins results in an autoimmune hypersensitivity with consequent production of antigen-antibody molecules [65]. These immune complexes accumulate on the vascular wall, especially in the microcirculation, causing damage to their thin layer of vascular endothelial cells, and resulting in widespread micro bleeding, a phenomenon known as disseminated intravascular coagulation (DIC). DIC has been associated with trypanosome infections in various host species [66–69] and has been observed in coatis infected with trypanosomes in the Pantanal region. Indeed, as observed here by path analysis, an increase in immune investment resulted in a worse body condition in ocelots and crab-eating foxes. DIC, together with the hypoferremic response discussed above, are the main causes of anemia observed in trypanosome-infected animals. Furthermore, oxidative stress due to oxidative damage in erythrocyte membranes are related to experimental and natural infection by *T*. *evansi* [70, 71].

## Conclusions

As *T*. *cruzi* is restricted to the New World, it had been interacting with its hosts over millions of years. On the contrary, *T*. *evansi* originates from the African continent, and has become a parasite of South American wild mammals only recently. In the Pantanal region, *T*. *evansi* was probably introduced together with horses and dogs in the late Eighteenth century when the first cattle farms were established. According to this scenario, while the course of *T*. *cruzi* infection is known to be predominantly chronic probably due to ancient association with its hosts, *T*. *evansi* infection of endemic Neotropical fauna may cause great damage to the health of its hosts, particularly due to increased virulence and pathogenicity of present interactions.

The anemia and immunosuppression evidenced by the present study, are associated with increasing habitat fragmentation and poaching [72], which poses a threat to wild coatis, ocelots and crab-eating foxes in the Pantanal wetland. Furthermore, due to epidemiological implications and conservation importance, studies of *T*. *cruzi* and *T*. *evansi* infections in free-living mammals should be a priority for health surveillance organizations, research promotion agencies, and postgraduate programs.

## Supporting information

S1 Fig Path analysis on anemia indicators of coatis (*Nasua nasua*). Results of path analysis on anemia indicators of coatis (*Nasua nasua*) infected with *Trypanosoma evansi* and coinfected with *T*. *evansi*/*Trypanosoma cruzi* in the sub-region of Nhecolândia, Pantanal, between November 2015 and October 2016.
(TIF)

S2 Fig Path analysis on infection responses of coatis (*Nasua nasua*). Results of path analysis on infection responses of coatis (*Nasua nasua*) infected with *Trypanosoma cruzi* and coinfected with *Trypanosoma evansi*/*T*. *cruzi* in the sub-region of Nhecolândia, Pantanal, between November 2015 and October 2016.
(TIF)

S3 Fig Path analysis on immune investment of coatis (*Nasua nasua*). Results of path analysis on immune investment of coatis (*Nasua nasua*) infected with *Trypanosoma cruzi* and coinfected with *Trypanosoma evansi*/*T*. *cruzi* in the sub-region of Nhecolândia, Pantanal, between November 2015 and October 2016.
(TIF)

S4 Fig Path analysis on infection responses of crab-eating foxes (*Cerdocyon thous*). Results of path analysis on infection responses of crab-eating foxes (*Cerdocyon thous*) infected with *Trypanosoma cruzi* and coinfected with *Trypanosoma evansi*/*T*. *cruzi* in the sub-region of Nhecolândia, Pantanal, between November 2015 and October 2016.
(TIF)

S5 Fig Path analysis on immune investment against *Trypanosoma cruzi* of crab-eating foxes (*Cerdocyon thous*). Results of path analysis on immune investment against *T*. *cruzi* in crab-eating foxes (*Cerdocyon thous*) infected with *Trypanosoma cruzi* and coinfected with *Trypanosoma evansi*/*T*. *cruzi* in the sub-region of Nhecolândia, Pantanal, between November 2015 and October 2016.
(TIF)

S6 Fig Path analysis on immune investment against *Trypanosoma evansi* of crab-eating foxes (*Cerdocyon thous*). Results of path analysis of immune investment against *T*. *evansi* in crab-eating foxes (*Cerdocyon thous*) infected with *Trypanosoma cruzi* and coinfected with *Trypanosoma evansi*/*T*. *cruzi* in the sub-region of Nhecolândia, Pantanal, between November 2015 and October 2016.
(TIF)

S7 Fig Path analysis on anemia indicators in ocelots (*Leopardus pardalis*). Results of path analysis on anemia indicators in ocelots (*Leopardus pardalis*) infected with *Trypanosoma evansi* and coinfected with *T*. *evansi*/*T*. *cruzi* in the sub-region of Nhecolândia, Pantanal, between November 2015 and October 2016.
(TIF)

S8 Fig Path analysis on immune investment against *Trypanosoma cruzi* of ocelots (*Leopardus pardalis*). Results of path analysis on immune investment against *Trypanosoma cruzi* in ocelots (*Leopardus pardalis*) infected with *T*. *evansi* and coinfected with *Trypanosoma evansi*/*T*. *cruzi* in the sub-region of Nhecolândia, Pantanal, between November 2015 and October 2016.
(TIF)

S9 Fig Path analysis on immune investment against *Trypanosoma evansi* of ocelots (*Leopardus pardalis*). Results of path analysis on immune investment against *Trypanosoma evansi* in ocelots (*Leopardus pardalis*) infected with *T*. *evansi* and coinfected with *T*. *evansi*/*Trypanosoma cruzi* in the sub-region of Nhecolândia, Pantanal, between November 2015 and October 2016.
(TIF)

S1 File Collection Data Set. Data of coatis (Nasua nasua), crab-eating-foxes (Cerdocyon thous) and ocelots (Leopardus pardalis) infected for Trypanosoma cruzi and Trypanosoma evansi in the Pantanal. Samples were collected from November 2015 up to October 2016.
(XLSX)
