# Correction: Outcomes of *Trypanosoma cruzi* and *Trypanosoma evansi* infections on health of Southern coati (*Nasua nasua*), crab-eating fox (*Cerdocyon thous*), and ocelot (*Leopardus pardalis*) in the Brazilian Pantanal

**DOI:** 10.1371/journal.pone.0205613

**Published:** 2018-10-17

**Authors:** Filipe Martins Santos, Gabriel Carvalho de Macedo, Wanessa Teixeira Gomes Barreto, Luiz Gustavo Rodrigues Oliveira-Santos, Carolina Martins Garcia, Guilherme de Miranda Mourão, Grasiela Edith de Oliveira Porfírio, Elizangela Domenis Marino, Marcos Rogério André, Lívia Perles, Carina Elisei de Oliveira, Gisele Braziliano de Andrade, Ana Maria Jansen, Heitor Miraglia Herrera

There are errors in the author abbreviations of the citation. Please see the correct citation here: Santos FM, Macedo GC, Barreto WTG, Oliveira-Santos LGR, Garcia CM, Mourão GM, et al. (2018) Outcomes of *Trypanosoma cruzi* and *Trypanosoma evansi* infections on health of Southern coati (*Nasua nasua*), crab-eating fox (*Cerdocyon thous*), and ocelot (*Leopardus pardalis*) in the Brazilian Pantanal. PLoS ONE 13(8): e0201357. https://doi.org/10.1371/journal.pone.0201357
