# Supplementary figures and images for: Outcomes of Trypanosoma cruzi and Trypanosoma evansi infections on health of Southern coati (Nasua nasua), crab-eating fox (Cerdocyon thous), and ocelot (Leopardus pardalis) in the Brazilian Pantanal
Source: PLoS One. 2018 Aug 15;13(8):e0201357. doi: 10.1371/journal.pone.0201357 (PMC6093643; doi:10.1371/journal.pone.0201357)

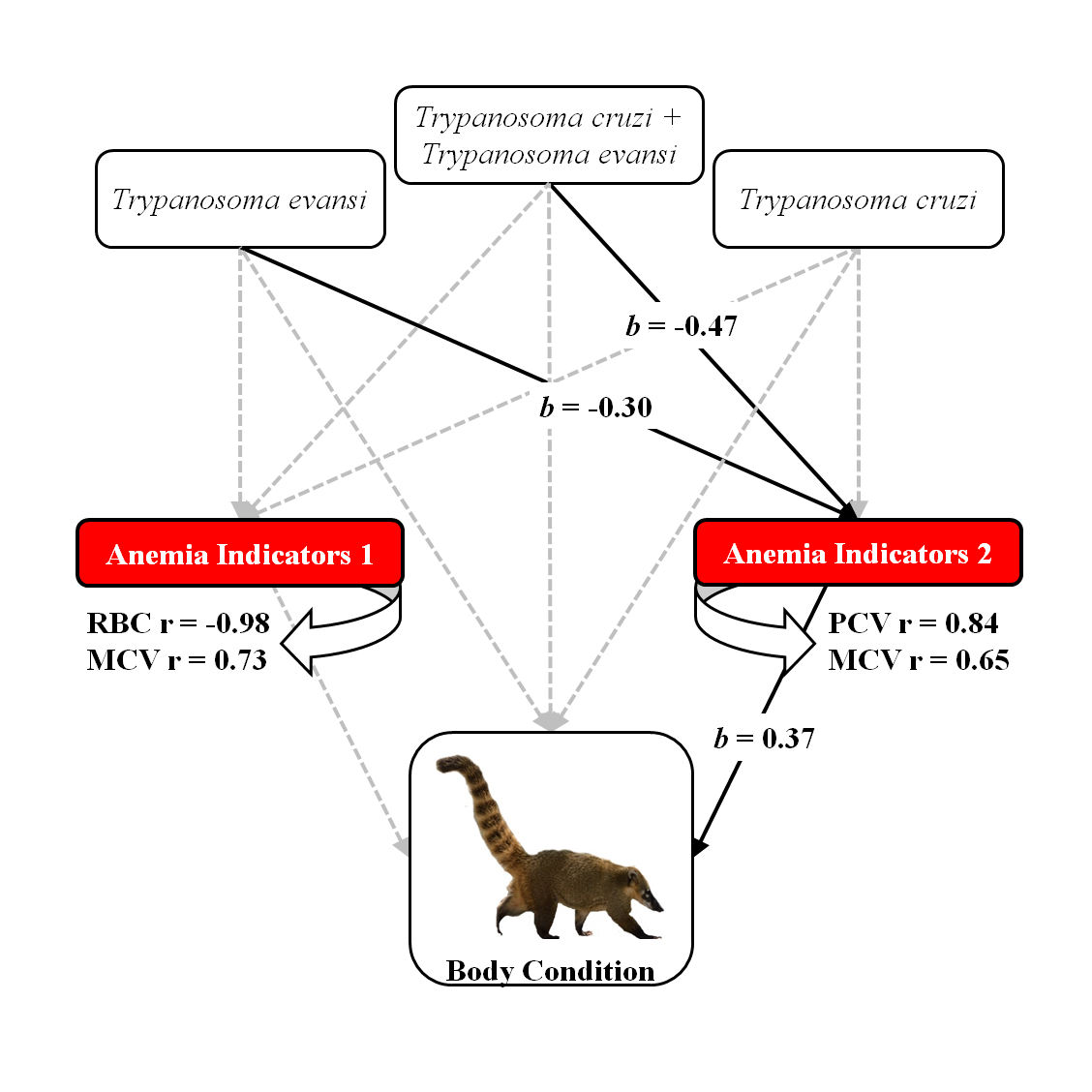

Supplement: S1 Fig — Results of path analysis on anemia indicators of coatis (Nasua nasua) infected with Trypanosoma evansi and coinfected with T. evansi/Trypanosoma cruzi in the sub-region of Nhecolândia, Pantanal, between November 2015 and October 2016. (TIF) [file pone.0201357.s001.tif]

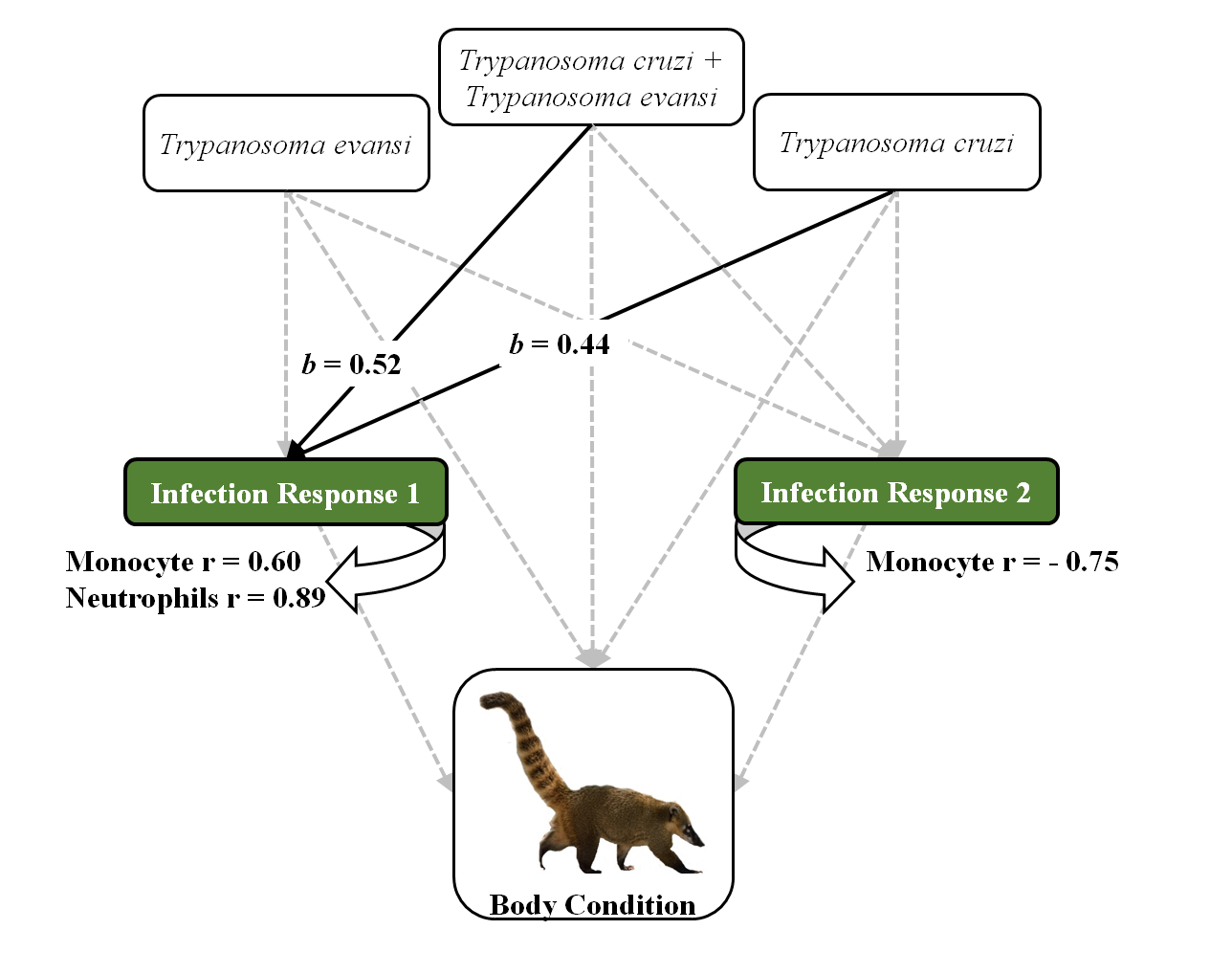

Supplement: S2 Fig — Results of path analysis on infection responses of coatis (Nasua nasua) infected with Trypanosoma cruzi and coinfected with Trypanosoma evansi/T. cruzi in the sub-region of Nhecolândia, Pantanal, between November 2015 and October 2016. (TIF) [file pone.0201357.s002.tif]

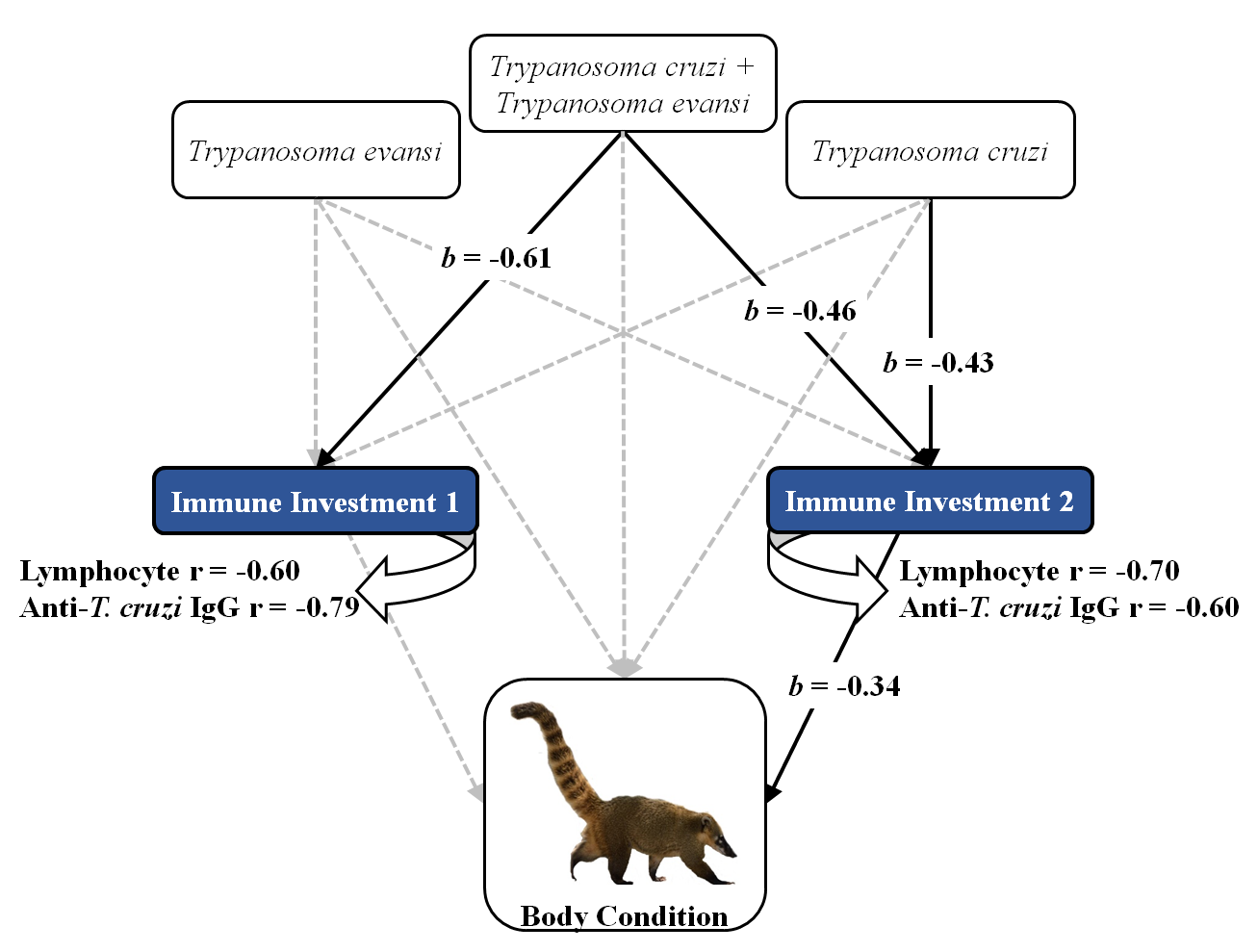

Supplement: S3 Fig — Results of path analysis on immune investment of coatis (Nasua nasua) infected with Trypanosoma cruzi and coinfected with Trypanosoma evansi/T. cruzi in the sub-region of Nhecolândia, Pantanal, between November 2015 and October 2016. (TIF) [file pone.0201357.s003.tif]

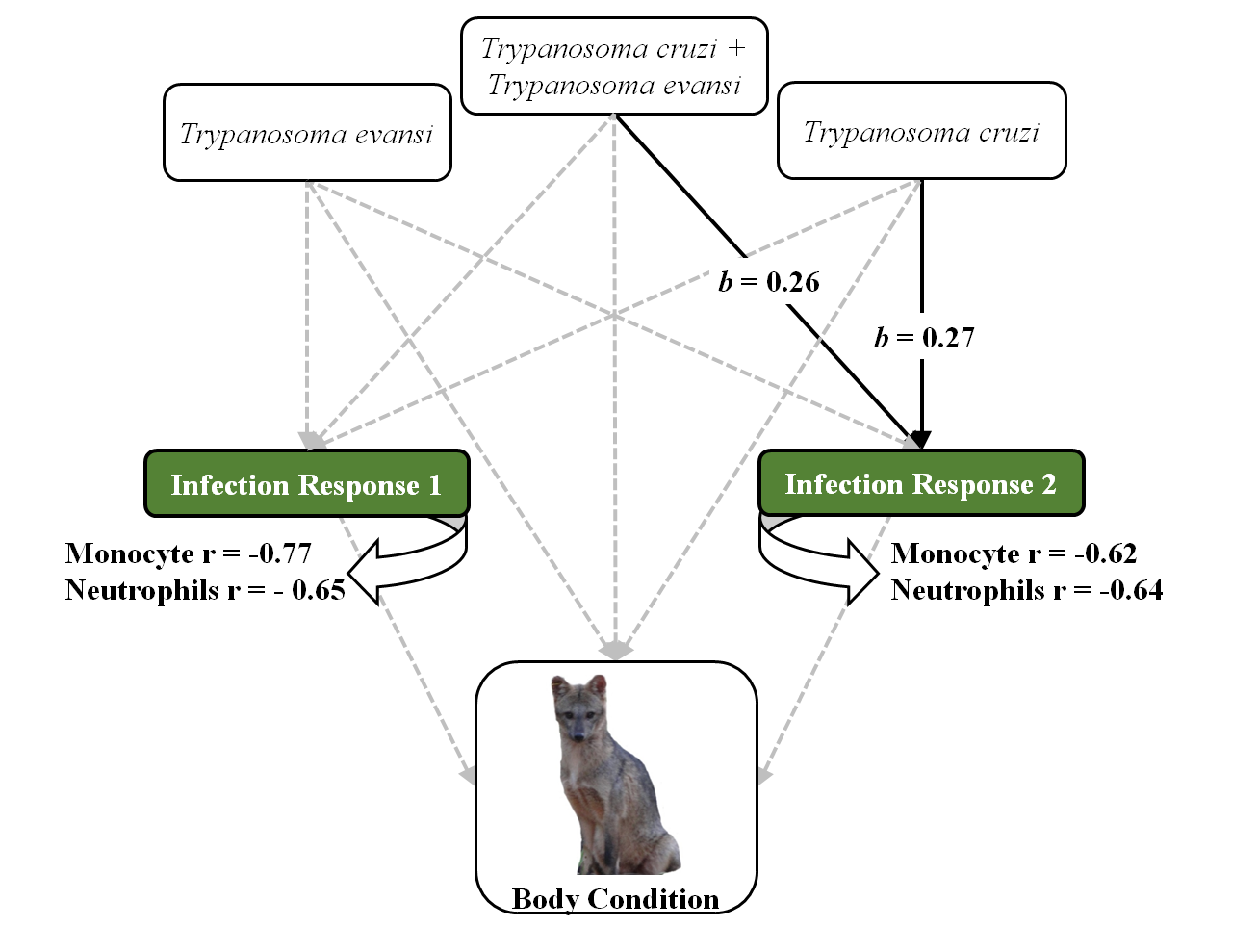

Supplement: S4 Fig — Results of path analysis on infection responses of crab-eating foxes (Cerdocyon thous) infected with Trypanosoma cruzi and coinfected with Trypanosoma evansi/T. cruzi in the sub-region of Nhecolândia, Pantanal, between November 2015 and October 2016. (TIF) [file pone.0201357.s004.tif]

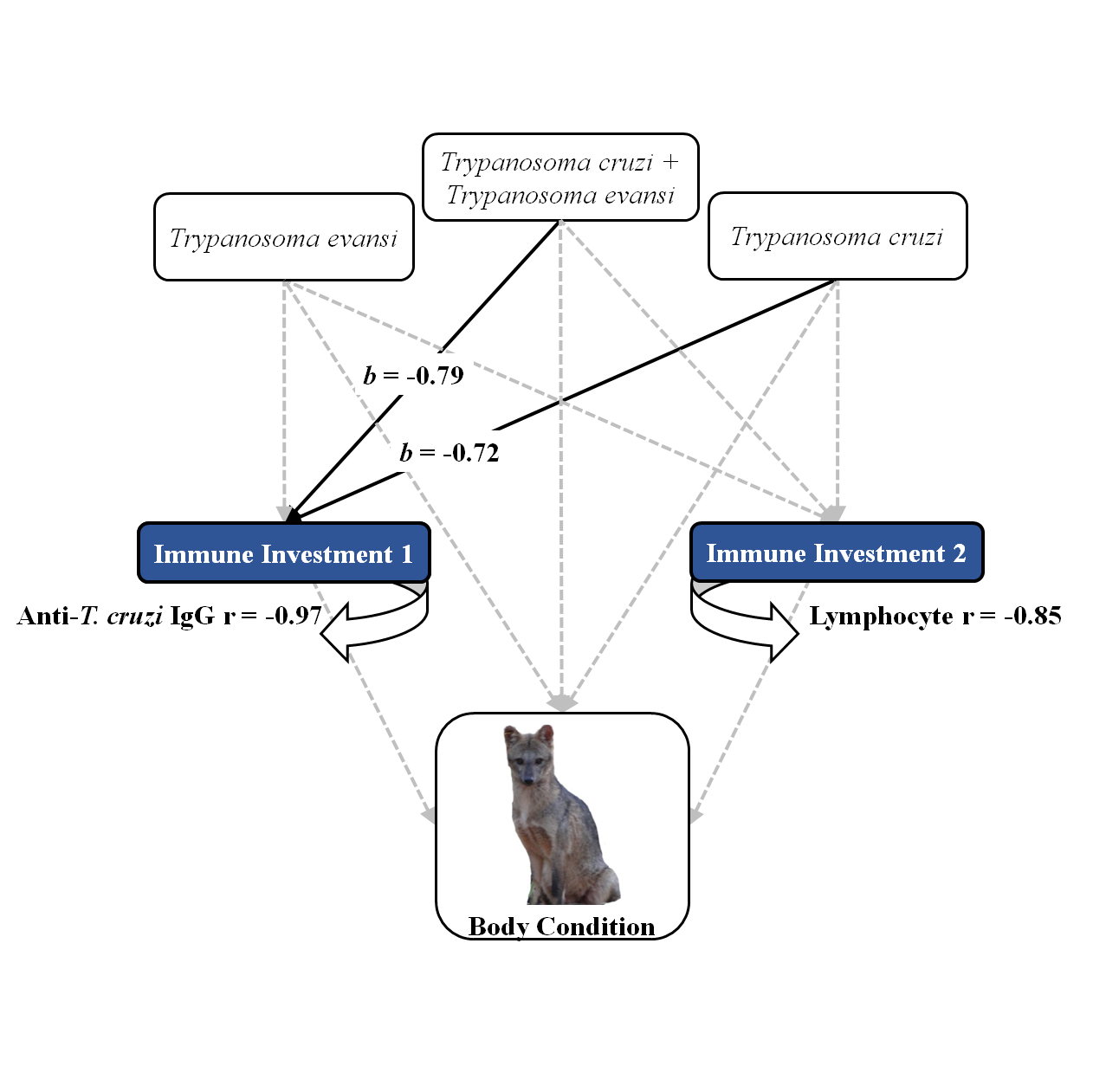

Supplement: S5 Fig — Results of path analysis on immune investment against T. cruzi in crab-eating foxes (Cerdocyon thous) infected with Trypanosoma cruzi and coinfected with Trypanosoma evansi/T. cruzi in the sub-region of Nhecolândia, Pantanal, between November 2015 and October 2016. (TIF) [file pone.0201357.s005.tif]

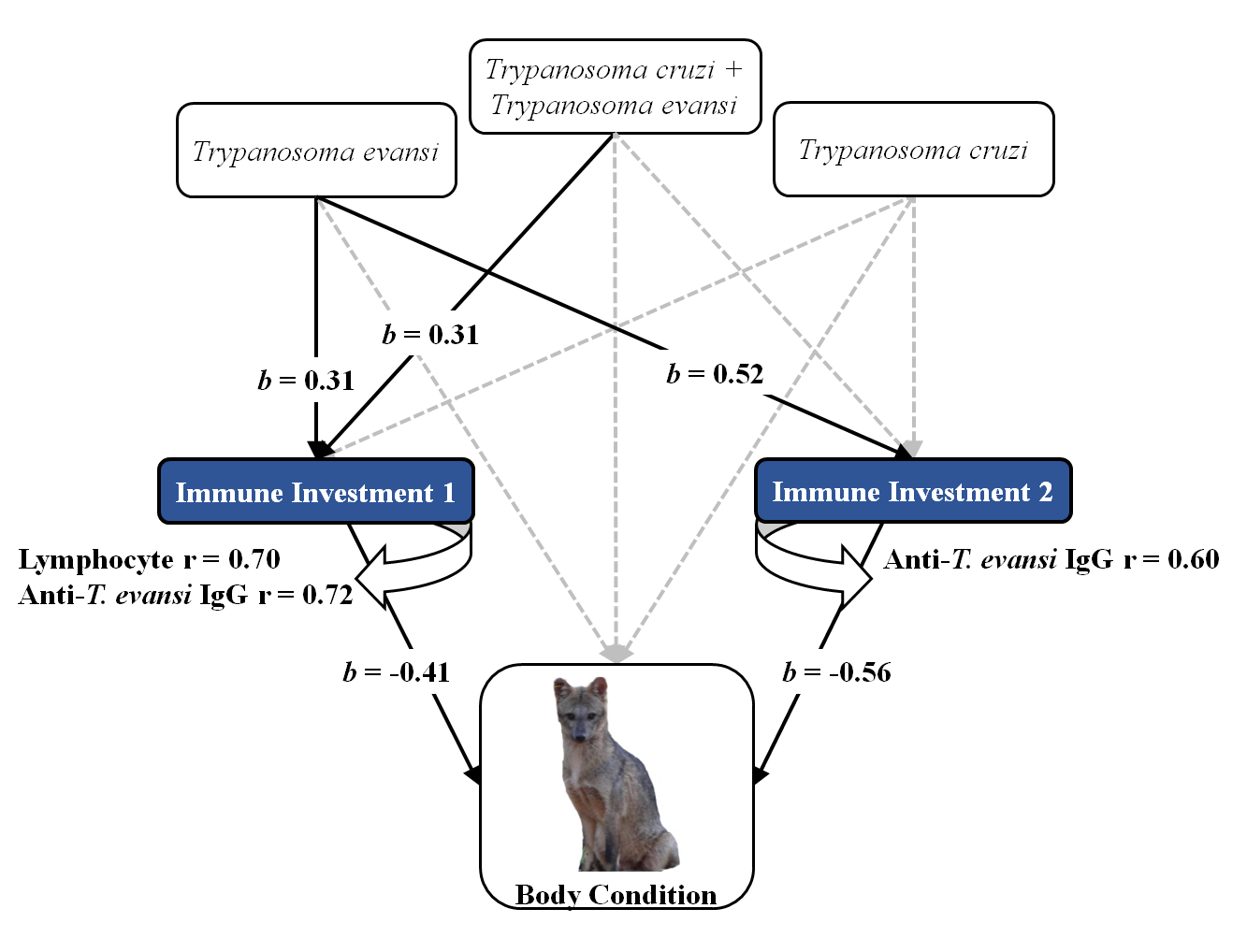

Supplement: S6 Fig — Results of path analysis of immune investment against T. evansi in crab-eating foxes (Cerdocyon thous) infected with Trypanosoma cruzi and coinfected with Trypanosoma evansi/T. cruzi in the sub-region of Nhecolândia, Pantanal, between November 2015 and October 2016. (TIF) [file pone.0201357.s006.tif]

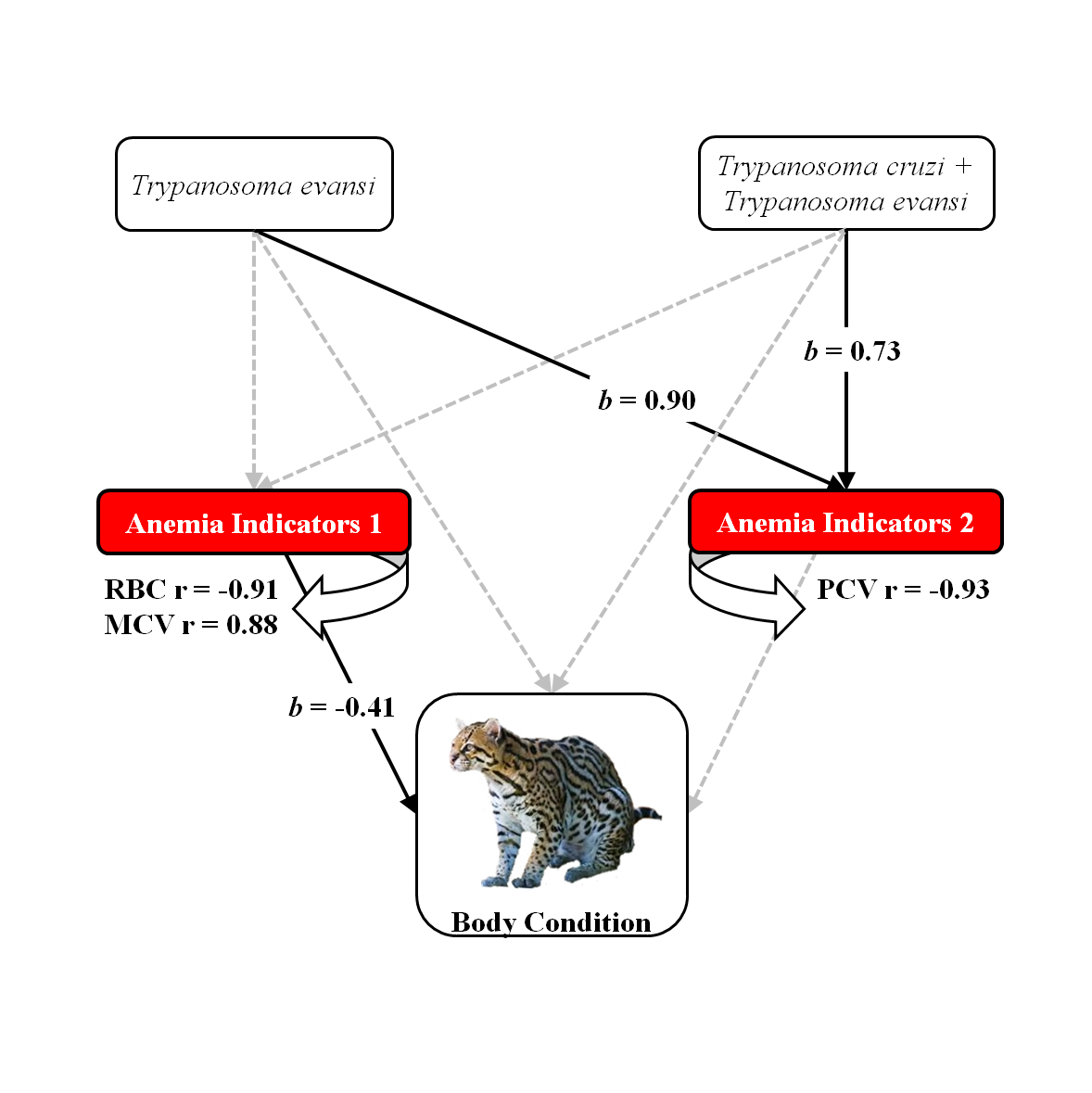

Supplement: S7 Fig — Results of path analysis on anemia indicators in ocelots (Leopardus pardalis) infected with Trypanosoma evansi and coinfected with T. evansi/T. cruzi in the sub-region of Nhecolândia, Pantanal, between November 2015 and October 2016. (TIF) [file pone.0201357.s007.tif]

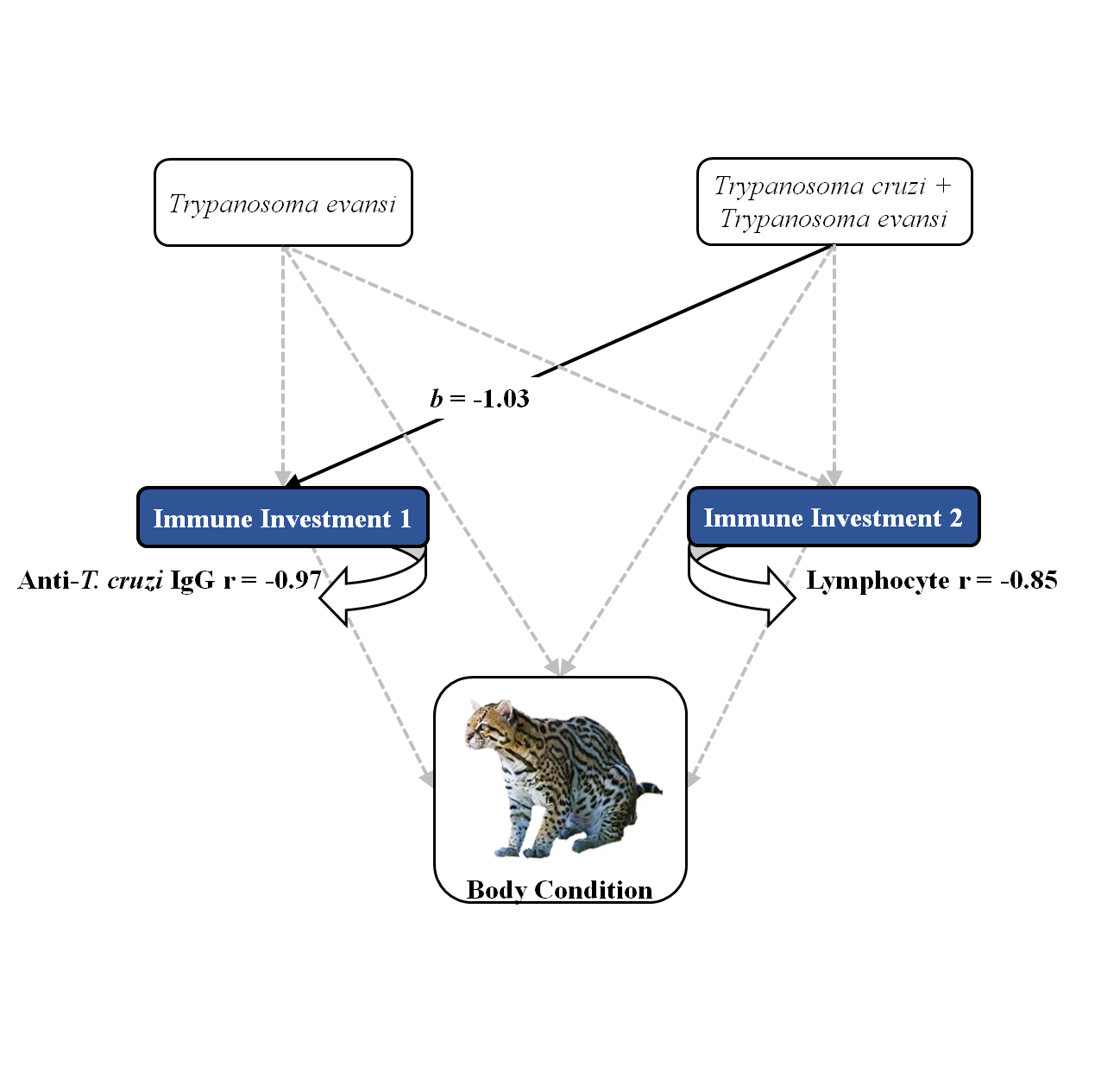

Supplement: S8 Fig — Results of path analysis on immune investment against Trypanosoma cruzi in ocelots (Leopardus pardalis) infected with T. evansi and coinfected with Trypanosoma evansi/T. cruzi in the sub-region of Nhecolândia, Pantanal, between November 2015 and October 2016. (TIF) [file pone.0201357.s008.tif]

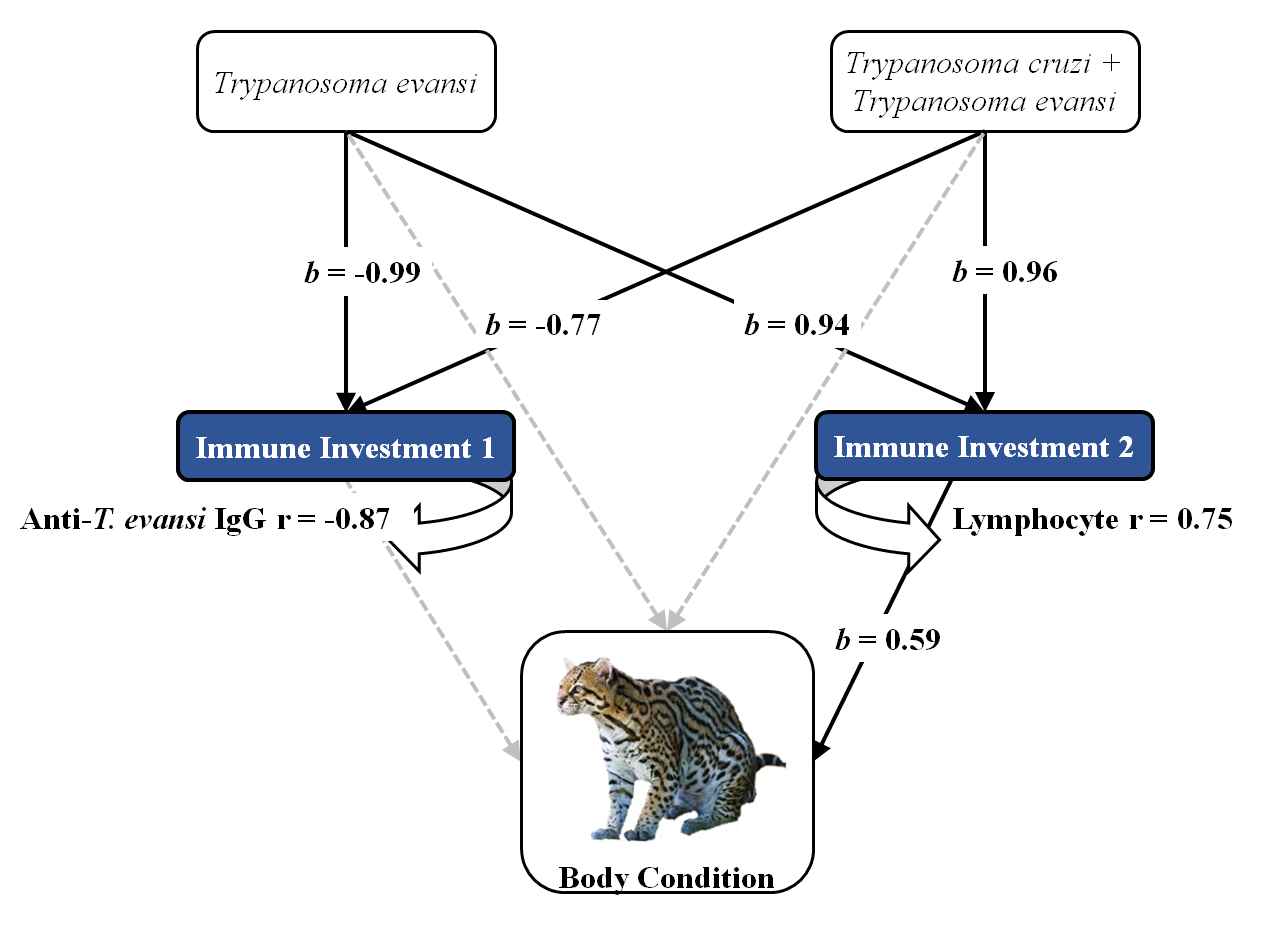

Supplement: S9 Fig — Results of path analysis on immune investment against Trypanosoma evansi in ocelots (Leopardus pardalis) infected with T. evansi and coinfected with T. evansi/Trypanosoma cruzi in the sub-region of Nhecolândia, Pantanal, between November 2015 and October 2016. (TIF) [file pone.0201357.s009.tif]
